# Positive selection of HIV host factors and the evolution of lentivirus genes

**DOI:** 10.1186/1471-2148-10-186

**Published:** 2010-06-18

**Authors:** Katarzyna Bożek, Thomas Lengauer

**Affiliations:** 1 Max Planck Institute for Informatics Computational Biology and Applied Algorithmics Campus E1 4 66123 SaarbrÃ¼cken, Germany

## Abstract

**Background:**

Positive selection of host proteins that interact with pathogens can indicate factors relevant for infection and potentially be a measure of pathogen driven evolution.

**Results:**

Our analysis of 1439 primate genes and 175 lentivirus genomes points to specific host factors of high genetic variability that could account for differences in susceptibility to disease and indicate specific mechanisms of host defense and pathogen adaptation. We find that the largest amount of genetic change occurs in genes coding for cellular membrane proteins of the host as well as in the viral envelope genes suggesting cell entry and immune evasion as the primary evolutionary interface between host and pathogen. We additionally detect the innate immune response as a gene functional group harboring large differences among primates that could potentially account for the different levels of immune activation in the HIV/SIV primate infection. We find a significant correlation between the evolutionary rates of interacting host and viral proteins pointing to processes of the host-pathogen biology that are relatively conserved among species and to those undergoing accelerated genetic evolution.

**Conclusions:**

These results indicate specific host factors and their functional groups experiencing pathogen driven evolutionary selection pressures. Individual host factors pointed to by our analysis might merit further study as potential targets of antiretroviral therapies.

## Background

Phylogenetic studies have shown that HIV emerged in humans through at least eleven cross-species transmission events of simian immunodeficiency virus (SIV) from non-human African primates. Three transmissions of chimpanzee SIV (SIVcpz) from the central African chimpanzee subspecies (*Pan troglodytes troglodytes*) gave rise to the HIV-1 groups M, N and O [1], with the group M causing the AIDS pandemic. Other transmissions of sooty mangabey (*Cercocebus torquatus atys*) SIV (SIVsmm) gave rise to HIV-2 groups A-H [2,3].

SIV infection of African non-human primate host species (including sooty mangabeys, African green monkeys, mandrills, and several others) is non-pathogenic despite high levels of viremia [4-6]. Different levels of pathogenicity of immunodeficiency viruses in their host species [4,7] as well as the lack of adaptation to their non-natural species [8,9] show how interspecies differences can impact viral infectivity and drive adaptation. Host genetic differences between individuals also affect the dynamics of disease progression [10]. There is a growing list of genes and alleles for which there is evidence of a positive or negative effect on infection and disease progression. Among them, several recently identified host factors block or restrict retroviral infections in primates: TRIM5α, a tripartite motif (TRIM) family protein [11]; apolipoprotein B editing catalytic polypeptide (APOBEC3G), a member of the family of cytidine deaminases [12] and tetherin (BST-2, CD317) [13]. These restriction factors constitute defense mechanisms of the host acting in a species-specific manner [8,9] blocking the viruses from replication in their non-natural host species and thus being potential agents of anti-HIV defense.

One feature of pathogenic HIV-1 infection that distinguishes it from non-pathogenic SIV infections is the high level of chronic immune activation associated with accelerated T-cell turnover rates and apoptosis [5]. The basis for this difference in pathogenicity is not understood, however deciphering which viral and host factors are responsible for the nonpathogenic course of natural SIV infections could prove useful in developing more effective treatments and prevention strategies for AIDS.

Positive selection, demonstrated in part by the rapidly evolving immune system genes [14], reflects the evolution of the host defense against various infections. Several HIV restriction factors have been shown to be under positive selection throughout primate evolution [15-18]. Due to the relatively long generation times of primate species with slow rate of genetic evolution in contrast to the short generation times of viruses with high rates of genetic evolution and the potentially recent introduction of SIV into primates [19], the impact of the selection pressures solely from SIV on the host species is likely to be negligible. However, genetic polymorphisms in genes interacting with the virus can influence traits relevant for the susceptibility to lentiviral infection and point to a potential role of a gene in infection and its contribution to disease. Comparative genomics can offer insights into disease mechanisms by correlating molecular differences that arose during primate evolution with the variation in disease susceptibility.

There is ample scientific knowledge on HIV-1 human protein interactions. The *HIV-1 Human Protein Interaction Database *is a catalogue of over 1400 human proteins that participate in approximately 3000 unique HIV-1-to-human protein interactions reported in peer reviewed scientific literature [20]. The size and scope of this database allow for large-scale analyses of HIV-host molecular interactions. Together with several fully sequenced primate genomes it allows for a systematic search for host factors under positive selection that might be relevant for infection and merit further investigation. Previous studies of positive selection in the HIV host factors focused on individual examples [15-18], as well as on a set of 140 proteins compiled from the literature [21]. Here we analyze all 1439 genes available in the *HIV-1 Human Protein Interaction Database*.

We explore genetic differences of HIV-interacting genes among primates. We perform a comparative genomics analysis of the HIV-interacting proteins in search of positively selected genes in four different primate species. We characterize the positively selected genes in terms of their biological function, role in the protein-protein interaction networks and interactions with the virus. We then analyze the relationship between the strength of positive selection in the host proteins with the evolutionary rates of the interacting proteins of five immunodeficiency viruses in search of patterns in the evolution of host-pathogen interactions.

## Results

### Positive selection

In order to find which of the 1439 host factors reported in the *HIV-1-Human Protein Interaction Database *are under positive selection we extracted and aligned their human genetic sequences with the respective homologs in three monkey species: chimpanzee, orangutan and rhesus macaque. We applied a measure of positive selection, based on the likelihood ratio test (LRT) for the presence of sites under positive selection. We considered a significant LRT to be an indicator of a gene being under positive selection. The number of positively selected sites in each gene sequence constitutes a *site-based score *of positive selection. In order to assess the robustness of, and provide additional support for, the site-based score we compared it with a *sliding window score *based on dN/dS ratio (non-synonymous to synonymous nucleotide substitution ratio) in sliding windows along the gene sequence.

In case of a discrepancy between the two positive selection scores we used the more stringent LRT significance as the indicator of positive selection, the sliding window measure was used as a supporting score. We used both tests to search for positive selection in all four primate species and subsequently in the human and chimpanzee gene sequences separately.

**Figure 1:**
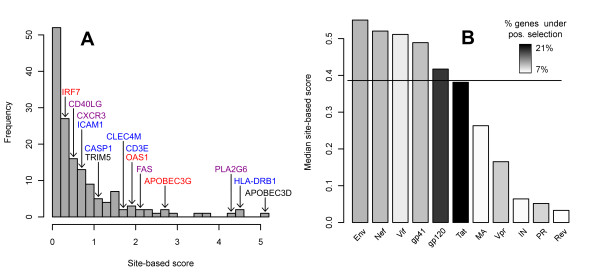
**Distribution of the site-based score in the genes of four primate species**. (A) Distribution of all gene scores. Positions of important host factors are indicated. Colors indicate the functional group to which the factors belong: blue - membrane-related proteins, red - innate immune response proteins, magenta - both, black - none of the groups. (B) Median values of site-based scores in the interaction grouping. Shown are groups of the size >2% of all interactions. Bar colors indicate the percentage of the genes under positive selection in each group as inferred by LRT (listed in Table 1). CA protein (not shown on the figure) has less that 1% interactions reported in the dataset showing a high median of 1.3.

The site-based search for genes under positive selection returned 152 genes having sites under positive selection among all four primate species and in 97 genes having sites under positive selection in the human-chimp comparison with an overlap of 49 genes. The full list of the analyzed genes together with all scores is provided in the Additional file 1. The genes were next ranked according to their site-based and sliding window scores. The ranks of genes obtained with the sliding window score correlate with the site-based score ranks with a correlation coefficient of 0.65 in all species and 0.52 in the human-chimp comparison (p < 0.01). This positive correlation of ranks obtained with two different methods, together with the high scores assigned to proteins reported to be under positive selection in other studies (APOBEC3G [16,18], TRIM5α [17] - Figure 1A) suggests that the ranks used in further analyses are robust with respect to the scoring method.

The recently identified host restriction factor tetherin (BST-2, CD317) [13] was not included in the *HIV-1 Human Interaction Database*. We separately extracted, aligned and performed the positive selection tests on the tetherin sequences of the four primate species. Even though positive selection in the primate tetherins has been reported before [15], the site-based approach did not result in significant LRT. However the sliding window test showed this protein to be under positive selection with a rank of 62 among the full list of 1182 genes (APOBEC3G rank 6, TRIM5α - 38).

In order to inspect the distribution of positive selection scores in subsets of the HIV-interacting genes we established an *interaction grouping *based on with which viral protein the host proteins interact. The interaction grouping showed variation in the distributions of site-based scores as well as in the ratio of positively selected host proteins among groups (Figure 1B). Permutation tests revealed a significantly lower mean of the ranks based on site-based scores of host genes interacting with gag protein and a comparatively higher mean for integrase (IN), protease (PR), vpr and rev proteins (Table 1). The ranks based on sliding window scores were additionally significantly lower for the envelope (Env)-, gp120-, gp41- and capsid (CA)-interacting genes and higher for Vif- matrix (MA)- and nucleocapsid (NC)-interacting genes. The site-based ranking was limited to the 152 genes under positive selection. The discrepancies of the significance of mean ranks of gene groups between the two scorings were due to the differing numbers of genes included in both rankings. The mean group ranks in both scorings are positively correlated with r = 0.52 (p ≈ 0.07). If we consider only the 152 genes under positive selection the correlation increases substantially to r = 0.92 (p < 0.05).

**Table 1:** Ranking of viral proteins based on the relative evolutionary rate and characterization of interacting host genes.

	rank	relative evol. rate	interacting genes	% genes under positive selection	mean norm. site-based rank	mean norm. sliding window rank
gp120	1	4.36	434	12.9	0.452	0.481^**(+)**^

Vpu	2	3.82	22	18.2	0.292	0.520

Env	3	3.06	145	15.9	0.448	0.497^**(+)**^

p6	4	2.43	12	16.7	0.258	0.681

Rev	5	2.21	59	6.8	0.737^**(-)**^	0.757^**(-)**^

gp41	6	2.07	123	16.3	0.427	0.488^**(+)**^

Nef	7	2.02	168	12.5	0.432	0.526

Gag	8	1.87	45	13.3	0.105^**(+)**^	0.615

Vif	9	1.77	55	16.4	0.350	0.659^**(-)**^

Tat	10	1.77	636	11.0	0.522	0.606

MA	11	1.70	69	7.2	0.542	0.742^**(-)**^

NC	12	1.60	19	15.8	0.416	0.759^**(-)**^

Vpr	13	1.58	152	11.2	0.647^**(-)**^	0.614

RT	14	1.42	33	9.1	0.606	0.621

Pol	15	1.38	1	100.0	0.190	0.094

CA	16	1.37	21	28.6	0.235	0.373^**(+)**^

PR	17	1.30	71	21.1	0.709^**(-)**^	0.629^**(-)**^

IN	18	1.00	66	7.6	0.729^**(-)**^	0.727^**(-)**^

Positively selected host genes are inferred from the significance of the LRT. Signs in parentheses indicate significantly high (+) and low (-) mean normalized rank of interacting host genes under positive selection. Ranks in both scorings are normalized to [0,1] range to facilitate comparison.

In order to additionally estimate positive selection that acted along the more recent timescale after the primate interspecies split we searched for single nucleotide polymorphisms (SNPs) in the human gene sequences. We found 61 genes containing positively selected SNPs with a maximum number of 5 SNPs in a gene (BRCA1) and the majority of genes having only one positively selected SNP (43 out of 61). Among genes found to be under positive selection using site-based method in the four primates and in the human-chimp comparison, 10 and 4 genes, respectively, were found to contain positively selected SNPs. We found no significant correlation between the presence of SNPs and positive selection in primates.

### Gene Ontology

Subsequently, we searched for Gene Ontology (GO) [22] terms enriched among the positively selected HIV-interacting genes. GO separates biological roles performed by genes of different organisms into three separate ontologies: *biological process*, *molecular function *and *cellular component*, each organized in a hierarchical manner with more general terms preceding more specific terms in the GO graph. We applied two statistical tests, Fisher's exact test (FET) and Kolmogorov-Smirnov test (KS), to test for the overrepresentation of terms in the three groups of positively selected genes: in all four primates, in human-chimp comparison and human only as inferred from the SNPs analysis resulting six enrichment tests in total. The FET and KS test for two different aspects of enrichment - enrichment of genes under positive selection and enrichment among high-ranking genes based on a positive selection score. The GO annotation of the genes from the HIV-1 Human Protein Interaction Database has been previously reported [23], our goal was to find which terms were specific for the three groups of genes being under positive selection as compared to the full set of HIV-interacting genes. The detailed results of all tests are provided as Additional file 2. Here we list selected terms that were significantly enriched (p < 0.05) in at least three out of the six tests.

**Figure 2:**
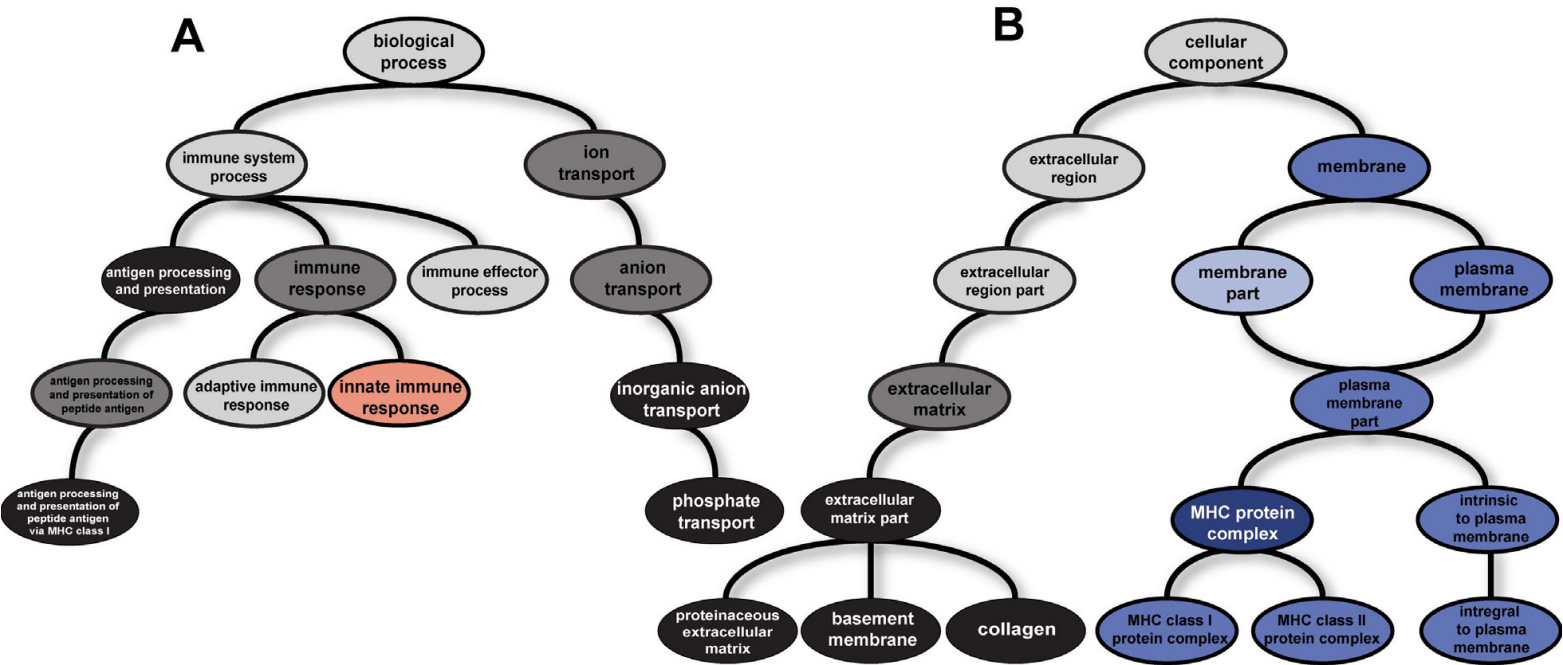
**GO graphs of terms of the biological process (A) and cellular component (B) ontologies enriched among the positively selected HIV-interacting genes**. Brightness of the nodes indicates the number of enrichment tests in which a term was found to be overrepresented (p < 0.05), ranging from three (light nodes) to five (darkest nodes) significant results out of six enrichments tests. The red node represents the innate immune response term, blue nodes represent membrane-related terms.

In the *biological process *ontology several terms related to immune response were found to be enriched among the positively selected genes (e.g. "antigen processing and presentation", "immune response" - Figure 2A) and several immune response terms were enriched among the high-ranking genes in all three groups of genes (e.g. "innate immune response" and "defense response to virus").

Among the terms of the *cellular component *ontology we found evidence of genes related to the cellular membrane being under positive selection. Eight out of 14 terms of this ontology that were overrepresented in at least four out of six enrichment tests were associated with the cellular membrane (Figure 2B). The MHC protein complex group of terms in this gene ontology also appeared to be enriched among positively selected HIV-interacting genes.

In the *molecular function *ontology the terms enriched in the positively selected genes are predominantly related to receptor or signaling activity, e.g. "receptor activity", "MHC class I and II activity", "transmembrane receptor activity", "signal transducer activity".

Given the high enrichment of the terms "membrane" and "innate immune response" we further analyzed genes in those two functional groups. To identify the first group we extracted all genes annotated with the GO term GO:0016020 ("membrane" from the *cellular component *ontology) and its subterms. To identify the innate immune response genes we performed a two step procedure. First, we extracted all genes annotated with the GO term GO:0045087 ("innate immune response" from the *biological process *ontology) and its subterms and then we searched our dataset for all proteins known to participate in the innate immune response [24,25].

We found the membrane-related proteins to be overrepresented among the positively selected genes in all four primates (chi-square test p < 0.05) and innate immune response to be overrepresented in all three groups of positively selected genes (chi-square test p < 0.001).

### Protein-protein interaction networks

To characterize the importance of the HIV-interacting proteins for the host and those among them being under positive selection, we analyzed their position in the network of human protein-protein interactions (PPIs) and human-pathogen PPIs, based on the analysis of human-pathogen interactions for 190 pathogens, both bacterial and viral, merged into 54 pathogen groups based on taxonomic similarity [26]. Dyer et al. [26] reported that both viral and bacterial proteins preferentially interact with human proteins that are either *hubs *- i.e., those involved in many interactions, or *bottlenecks *- i.e., those central to many human PPI network pathways suggesting that pathogens may have evolved to interact with proteins controlling critical processes in the host cell as a mechanism of disrupting the key elements of the host cellular machinery. We inspected the distribution of the local connectivity, centrality, number of interacting pathogens, and number of pathogen groups for the host proteins in the interaction grouping. Local connectivity of a protein is defined as the number of human PPIs in which it participates; centrality is the fraction of shortest paths in human PPI network between all protein pairs that pass through the given protein. High centrality is characteristic of a bottleneck in an interaction network, high connectivity - of a hub.

We found that IN, p6, PR, Rev, reverse transcriptase (RT), Vif, Vpr interact with host proteins of a significantly high local connectivity. p6 and Rev interact with proteins that are also highly central (Table 2). In contrast, gp41, Env, CA and Gag seem to interact with host proteins of a significantly lower local connectivity.

**Table 2:** Interaction specificities of HIV-related host factors in the interaction grouping.

		proteins	local connectivity	centrality	pathogens	pathogen groups
	PR	27	<0.01 (+)	<0.01 (+)	-	-
	
Pol-encoded	RT	20	0.01 (+)	<0.01 (+)	-	-
	
	IN	49	<0.01 (+)	-	-	<0.01 (+)

	Nef	97	-	0.03 (+)	-	-

	Rev	42	<0.01 (+)	0.01 (+)	<0.01 (+)	<0.01 (+)

	Tat	455	-	-	-	0.01 (-)

	Vif	45	<0.01 (+)	-	-	<0.01 (+)

	Vpr	98	<0.01 (+)	0.03 (+)	0.03 (+)	0.01 (+)

	Vpu	20	-	<0.01 (+)	0.02 (+)	0.01 (+)

	MA	54	-	-	-	<0.01 (+)
	
	CA	18	0.03 (-)	-	-	-
Gag-encoded						
	NC	16	-	-	<0.01 (+)	-
	
	p6	8	0.01 (+)	<0.01 (+)	<0.01 (+)	<0.01 (+)
	
	Gag	26	0.01 (-)	-	0.01 (-)	<0.01 (+)

	gp120	175	-	-	-	-
	
	gp41	61	<0.01 (-)	-	0.01 (-)	<0.01 (-)
Env-encoded						
	Env	57	0.04 (-)	-	-	-

P-values of the significance of local connectivity, centrality, number of interacting pathogens and pathogen groups are indicated with a (+) sign for high values and a (-) sign for low values.

Given the low local connectivity of envelope-interacting host proteins, we investigated the distributions of the local connectivity, centrality, number of interacting pathogens and pathogen groups of the HIV-interacting proteins annotated with membrane-related GO terms. These proteins were of 0.73-fold lower degree (p < 0.01), interact with 0.69-fold lower number of pathogens (p < 0.01) and 0.93-fold lower number pathogen groups (p < 0.03).

All of the positively selected genes in the four primates tend to be less connected (0.76-fold difference, p ≈ 0.05) and less centrally located (0.74-fold difference, not significant, p ≈ 0.16) in the human PPI than the genes that show no positive selection. No clear patterns were observed in the number of interacting pathogens and pathogen groups or in the positively selected genes in the human-chimp comparison.

### Virus evolutionary rates

In order to further characterize the host proteins interacting with the virus, we devised a measure of genetic variability in the viral genes of immunodeficiency virus species corresponding to the positive selection measure in the primate genes. We aligned 175 genomes of five species of HIVs and SIVs and the genomes of the HIV-1 and SIVcpz only (HIV-1/SIVcpz alignment). Viral gene sequences extracted from the alignments were ranked according to their *relative evolutionary rates *(Table 1), a measure of genetic variability in the genes based on their phylogenetic distances.

In order to determine how this ranking depended on the distance measure, we recalculated the relative evolutionary rates based on Hamming distances. The ranks based on distances inferred from phylogenetic trees correlated with the rates based on Hamming distance (r = 0.93, p < 0.001 in the alignment of all viruses and r = 0.94, p < 0.001 in the HIV-1/SIVcpz alignment). The main goal of the ranking was to compare the relative variability of the viral genes in order to assess the correlation with the positive selection of the interacting host genes. Regardless of the distance measure used in both of the alignments, Env and its two subproteins together with Vpu and Rev were among the top 6 in the protein ranking. Polymerase (Pol) and Pol-encoded proteins (RT, PR, IN) together with CA were among the 5 lowest ranked proteins. The only discordance among the distance measures and alignments were observed among the middle-ranked genes (Nef, Gag, Vif, Tat, MA, NC, Vpr, RT).

### Gene rank correlation

To investigate the relative rates of evolution among the interacting host and virus genes we next calculated the correlation between the strength of positive selection acting on the host factors and the rate of evolution of the interacting viral genes. We calculated the correlation of the host factor ranks assigned according to the site-based and the sliding window scores and the viral gene ranks assigned according to the relative evolutionary rate measure. The ranks of interacting host and viral proteins showed only minor but significant (p < 0.01) positive correlation (r = 0.18 in the site-based and r = 0.17 in the sliding window scoring). In the human-chimp comparison the sliding window ranking produced the only significant correlation (r = 0.14, p < 0.01).

**Figure 3:**
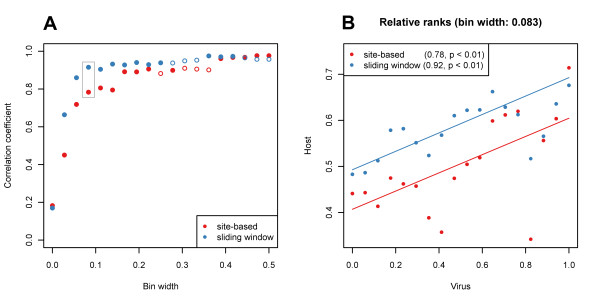
**Correlation of the normalized ranks of the interacting host and viral proteins obtained from site-based and sliding window scoring of the host genes and relative evolutionary rates of the viral genes in the comparison of the all four primate species and the alignment of all viruses**. (A) Correlation coefficients of the ranks of interacting proteins averaged by bin size. Significant correlation coefficients (p ≤ 0.05) are indicated by filled dots, the gray box indicates correlation coefficients of ranks plotted in panel B. (B) Plot of viral and host binned ranks. The correlation coefficients obtained for each host gene scoring is indicated in the brackets, lines are fitted according to least sum of squares.

Given the small but significant correlation of the ranks of interacting host and virus proteins we devised an *interaction binning *test to investigate the proximate relationships between interacting gene ranks. Bins were defined on the viral gene ranking, the ranks of host genes within one bin were averaged. 18 bin sizes were tested containing all possible number of genes adjacent in the viral gene ranking. The test showed a markedly increased correlation of approximate ranks of interacting viral and host genes (Figure 3A). For example, binning interactions over a bin of size 0.083, that averages host gene ranks interacting with 3 viral genes neighboring in the viral gene ranking increases substantially the correlation to r = 0.78 in the site-based and r = 0.92 in the sliding window scoring (Figure 3B). Permutation tests showed the significant correlations (p ≤ 0.05) in 14/18 bin sizes in the site-based ranking and in 13/18 in the sliding window ranking. In the human-chimp comparison only the correlation based on the sliding window score was significant for 16/18 bin sizes, with the correlation of 0.93 on average and of 0.88 for the 0.083 bin size. The high correlations obtained after binning suggest that the viral genes of different evolutionary rates tend to interact with host factors under commensurate levels of positive selection.

## Discussion and conclusions

Understanding the genome-wide selection pressure on HIV-interacting proteins can provide insights into the evolutionary dynamics of host factors, the genetic basis of differences between nonpathogenic and pathogenic lentivirus infection and the roles of individual genes in host-pathogen interaction and immunopathogenesis. Only two of the host species analyzed in this study are naturally infected with HIV/SIV (human and chimpanzee) moreover because these viruses have been recently introduced into their host species [7] the selection pressures we observe are not driven by modern lentiviruses.

Even though the interaction data used is mainly human and HIV-1 specific, the majority of interactions are shared with SIVs and with many other viruses. Many of the host factors found to be under positive selection interact with multiple pathogens so the selection pressures on the host factors are likely to be driven by those pathogens. However the comprehensive HIV interaction data offers opportunity for a broad study of the evolution of host-pathogen interactions. The interactions in the *HIV-1 Human Protein Interaction Database *are catalogued manually based on a literature screen and cannot be considered as fully validated. Nevertheless our screen for positive selection points to a narrow set of potentially interesting interactions that can be examined and validated individually.

In our analysis we identify ~10% of the 1439 HIV-interacting genes as being under positive selection based on LRT of sites under positive selection in four primate species. Ortiz et al. [21] reported a similar fraction of genes as being under positive selection based on the analysis of 140 genes. Among the 62 proteins analyzed in both this study and by Ortiz et al. 13 are identified as being under positive selection in the Ortiz et al. study. Among these, 11 are confirmed in our study either by showing significant LRT or being within the upper quintile of the sliding window scores. The reasons for discrepancies between the two studies might lie in different criteria of which genes to analyze and an expanded number of species for the analysis of individual genes in the Ortiz et al. study.

Three screens using small interfering RNA (siRNA) have been reported [27-29] that search human genes having effect on HIV infection - so called HIV dependency factors (HDFs). The overlap among the HDFs identified in the three studies and the proteins in the HIV interaction database is known to be small [30]. We scanned the results of one of the siRNA screening studies [27] for the presence of genes under positive selection detected in our analysis. Among 32 genes common between ours and Brass et al. studies only one protein (SP110) appeared to be under positive selection.

Several host factors not identified as being under positive selection in the LRT showed an elevated dN/dS ratio in the sliding window test. For example, tetherin, previously reported to be under positive selection [15], did not show significant LRT but ranked among the top 5% of all HIV-interacting proteins in the sliding window test. However, in order to be able to identify genes with small number of polymorphic sites in relatively long and conserved sequences we relied on the criterion of the significance of the LRT to assess positive selection.

**Figure 4:**
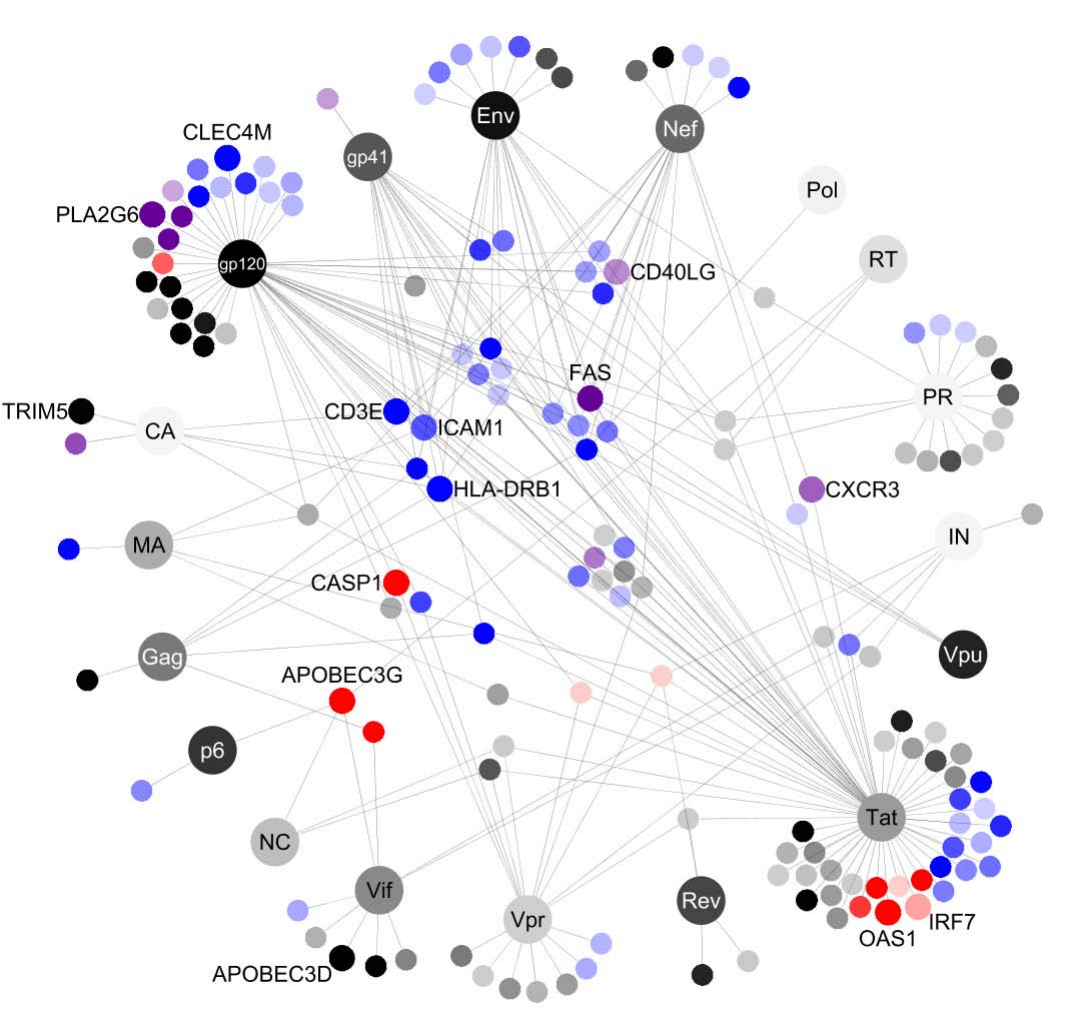
**HIV-human protein interaction network**. Viral proteins are represented by the largest dots. The lightness of each dot indicates the rank of relative evolutionary rates of the viral proteins with dark colors representing a high rank and light colors representing a low rank. Host proteins are represented by smaller dots positioned among the interacting viral proteins. Nodes of the host factors discussed in the text are enlarged. Colors indicate the functional group to which the factors belong: blue - membrane-related proteins, red - innate immune response proteins, magenta - both. The intensity of the colors indicates the positive selection score in the site-based scoring with stronger colors representing a high rank and less intensive colors representing a low rank. Only host proteins with significant LRT in the comparison of the four primates are shown. The network was rendered using Cytoscape [77].

Our functional analysis of the HIV-interacting host factors under positive selection pointed to two functional groups showing evidence of positive selection: membrane-related proteins and innate immune response proteins. These functional groups were overrepresented among the subsets of genes positively selected in all four primates, in human-chimp comparison and human only as inferred from the SNPs analysis (Figure 2). The interaction network of the virus proteins and the positively selected host proteins discussed below is visualized in Figure 4.

Among the membrane-related HIV-interacting proteins under positive selection we observe several that are known to interact with other pathogens. C-type lectin (CLEC4M) a transmembrane receptor expressed on the surface of dendritic cells and macrophages, known to bind gp120 [31], is a part of signaling pathways induced by other pathogens such as *Mycobacterium tuberculosis *[32], *hepatitis C virus *[33] and *ebola *virus [34]. Intercellular adhesion molecule 1 (ICAM1), a cell surface glycoprotein expressed in endothelial cells and cells of the immune system involved in a range of interactions with the HIV [35-38] is a receptor used by the *rhinovirus *[39]. Chemokine (C-X-C motif) receptor 3 (CXCR3), a G-protein-coupled receptor expressed in activated T cells, NK cells, and dendritic cells, suggested to interact with Nef [40] and Tat [41], participates in the signaling cascade of the T-cell activation in *genital herpes simplex virus type 2 *[42] and *hepatitis C virus *[43] infections. Binding to the surface receptors of the host cell and internalization into that cell are the first steps of the viral infection; the genetic variability of the proteins expressed on the cell surface therefore represents a potential host defense mechanism to infection, preventing viral recognition and efficient cell entry. The genetic variation in membrane genes has been previously reported [44]. This variation might have resulted from previous infections and can have impact on modern lentivirus restriction and primate species susceptibility to infection.

We detected a substantial amount of genetic variation in the membrane gene CD3E (rank 17 in the site-based, 3 in the sliding window score), coding for a part of the T-cell receptor CD3 complex (CD3-TCR). The highest concentration of positively selected residues was found in the protein extracellular and transmembrane domains. The CD3-TCR complex is known to participate in the mechanism responsible for different levels of immune activation and T-cell apoptosis in pathogenic HIV-human and nonpathogenic SIV-monkey infections [45,46]. This and other examples of membrane proteins being under positive selection suggests that other proteins in this functional group might be relevant for differences in HIV pathogenesis.

Similar to the membrane-related genes, we find the innate immune response gene group to be significantly enriched among the positively selected genes. The innate immune response is the first line of defense against viral infection detecting virus particles, modulating signaling pathways leading to an increased interferon (IFN) production and inhibition of virus spread to limit pathogenesis. There is growing evidence that the variability in the innate immune response proteins among primate species might contribute to different levels of immune activation in immunodeficiency virus infections [21,47].

We found interferon regulatory factor 7 (IRF7) to be under positive selection both in the test of four primate species and in the human-chimp comparison. IRF7 is a transcriptional factor involved in signaling triggered by the Toll-like receptor (TLR) TLR7 and TLR9 in response to the SIV infection [47]. Previous studies have shown that the genetic change in the primate IRF7 is correlated with the immune activation in SIV infection of primates [47]. Mutations in IRF7 might therefore be responsible for an altered TLR7 and TLR9 signaling leading to different levels of immune activation between pathogenic and nonpathogenic infections.

Caspase 1 (CASP1), a member of cysteine-aspartic acid protease family is an example of innate immune response gene under positive selection in primates (rank 31 in site-based and 26 in sliding window scoring) that is also located on a positively selected allele in humans. CASP1 is known to play a role in cellular apoptosis induced by HIV-1 Vpr [48] and gp120 [49]. Caspase activation has also been shown to participate in the cellular defense against *cowpox *[50], *baculovirus *[51], and *dengue virus *[52]. HIV-1 interaction with the caspase-regulated apoptosis might play a role in limiting the host immune response to the virus and facilitating viral persistence.

Among the high-scoring innate immune response genes under positive selection we also found Fas (APO-1, CD95) (rank 13 in the site-based and 46 in the sliding window scoring), a member of the tumor necrosis factor (TNF) receptor family. The Fas-mediated pathway plays an important role in HIV-1 immunopathogenesis. Fas has been suggested to contribute to the loss of CD4^+ ^T-cells in progression to AIDS as a part of the TCR-CD3 signaling pathway [53,54]. High levels of positive selection in Fas among primates and its regulatory relationship with TCR-CD3 point to this molecular pathway being possibly differently regulated among primates and partially responsible for immunopathogenesis.

Hyperactivation of the immune system in response to a pathogen recently introduced from another species can result in severe pathogenesis (e.g. hantavirus [55]). Disentangling the interaction of the HIV with the innate immune response and the host-specific differences in primate species might thus be of therapeutic interest. The examples of innate immune response genes under positive selection relevant for diverse immune activation in HIV infection suggest that other genes reported by our analysis might merit further study.

To gain further insight into the positively selected genes we grouped them according to their interacting viral genes and characterized each group with the use of the positive selection score, gene roles in the PPI network and the evolutionary rate of the corresponding viral gene. Among the gene groups, genes interacting with the viral envelope and its subunits show elevated scores of positive selection. In contrast, we found limited evidence of positive selection in the genes interacting with viral Pol-encoded genes, with PR and IN in particular.

In the context of their role in the PPI network and interactions with other pathogens the envelope and gp41-interacting genes revealed a significantly low local connectivity, centrality and number of interacting pathogens and pathogen groups. The same pattern was observed for the group of membrane-related genes. This less crucial role in the PPI and high specificity for the interacting pathogens might be key factors allowing for the accelerated evolution of the membrane and envelope-interacting genes. The less critical role of these proteins in the host biology makes them potential drug targets, for example, the chemokine receptor CCR5 is targeted by the Maraviroc antagonist [56].

In contrast to the host proteins interacting with the viral envelope and its subunits we found significantly higher local connectivity and centrality in human PPIs of the host genes interacting with viral Pol-encoded genes. Together with their higher conservation this suggests that the crucial role of these highly connected genes in host cellular biology could be an evolutionary constraint. Pathogens might therefore interact preferentially with highly connected human proteins not only as a strategy to control critical host molecular processes [26] but also because the lack of variation makes them a static target for virus-host interaction.

The high variability of the viral envelope gene and its subunits gp120 and gp41 [57,58] is in accordance with the high positive selection score of envelope-interacting and host membrane genes in general. Envelope proteins that participate in the crucial steps of binding to the cell receptors and coreceptors and membrane fusion [59,60], are located on the surface of the virion and contain recognition sites for various adaptive immune responses. Thus changes in optimal host cell receptor affinity and evasion of host immune responses create selection pressure on the Env gene. Together with the positive selection acting on the host membrane genes it points to the viral recognition and host cell receptor affinity as processes in which both the viral and host genes undergo accelerated evolution driven by viral evasion and host suppression.

Conversely, Pol-encoded proteins showed the least genetic variation among viral proteins. These proteins perform essential enzymatic functions common to all retroviruses, such as RNA retrotranscription, DNA integration and protein maturation and are unsurprisingly among the most conserved HIV/SIV proteins. Host factors interacting with the Pol-encoded proteins also show less genetic variation among the HIV-interacting genes, suggesting that these interactions tend to be the conserved parts of the host-pathogen interface.

The significant correlation of the viral and host gene ranks based on the rates of gene evolution further supports the hypothesis of reciprocal evolutionary effects between interacting host and pathogen proteins [61]. Highly conserved processes tend to be those crucial for viral replication; those less conserved might not be essential for the virus survival but involve accessory proteins and might contribute to pathogenesis. The highest rates of genetic change tend to be in the processes acting on the viral envelope and host cell membrane as they involve viral evasion and host pathogen immune recognition.

We identified and characterized host defense factors under positive selection potentially involved in different responses to immunodeficiency virus infections in primates. Identifying genetic differences in the interacting proteins can open the way to biological testing of hypotheses regarding their role in various SIV/HIV infection phenotypes with different levels of immune activation. In addition to providing new insights into viral pathogenesis and host immunity, the approach presented here provides the potential for discovering new targets for antiviral therapies based on the knowledge of crucial elements of the host-pathogen interface and the pace of their evolution.

## Methods

### Primate sequence analysis

#### Sequences

From the University of California, Santa Cruz (UCSC) *Genome Browser Database *(GBD) [62] we extracted the gene sequences of human proteins reported in the *HIV-1 Human Interaction Database *[20] to interact with HIV-1 as well as of the respective homologs in three non-human primate species: chimpanzee (*Pan troglodytes*), orangutan (*Pongo pygmaeus abelii*) and rhesus macaque *(Macaca mulatta)*. We used all available primate species for which genome sequences were publicly available and at least partially annotated. Homologous sequences were aligned using *Threaded Blockset Aligner *(TBA) [63] with the human sequence as the reference and trimmed to their coding parts based on the human gene annotation. We excluded sequences of genes not identified in the human genome or in more than one non-human primate species. We also excluded sequences composed of >50% gaps as compared to the human sequence. This filter helped to ensure that the results of the analysis are not influenced by the lack of proper homolog identification. The information on the alignment quality is provided in Additional file 3. Of the 1439 human proteins and 3643 unique interactions in the interaction database, 1182 proteins involved in 2596 unique interactions fulfilled our alignment criteria.

#### Positive selection in primate species

The site-based score was based on the *Bayes Empirical Bayes *(BEB) approach for inference of amino acids under positive selection [64] as implemented in the PAML package [65]. This approach uses a statistical distribution to describe the variation of the dN/dS ratio among sites and a LRT to compare two distributions: a distribution that allows a subset of sites to have dN/dS >1 and a null model that does not. If the result of the LRT is statistically significant then one can infer a gene to be under positive selection. An empirical Bayes test is then used to calculate the probability that a site belongs to the class of sites whose dN/dS ratio is larger than 1. We applied two LRTs implemented in the PAML package: M1a (NearlyNeutral) to M2a (PositiveSelection) comparison and M7 (beta) to M8 (beta&ω) comparison retaining the results of the higher LRT. The first LRT compares the NearlyNeutral null model which assumes two site classes one with 0 < dN/dS < 1 and one with dN/dS = 1 to the alternative model PositiveSelection which adds a site class of dN/dS >1. The second LRT compares the beta null model which assumes a beta distribution for dN/dS in the interval (0,1) to the alternative model beta&ω which adds a site class with dN/dS >1. The site-based score is based on the weighted sum of sites with dN/dS >1 normalized by the sequence length. We weighted these sites according to the calculated probability P of a site being under positive selection: by a factor of 3 for the sites with P > 0.99, a factor of 2 for the sites with P > 0.95, other sites by a factor of 1.

The sliding window score was based on the dN/dS ratio averaged over each sliding window across the protein gene sequence. We used the method of estimating the dN/dS substitution rates of entire sequences by Yang and Nielsen [66] also from the PAML package. The calculation of the dN/dS ratio was done in windows of 150 base-pair length (50 amino acids) slid along the sequence by a step of 30 base-pairs (10 amino acids). This test facilitates the localization of regions with a high dN/dS ratio in gene sequences rather than specific sites returned by the BEB approach.

While there are potential false positives associated with the LRT some genes might be missed by the sliding window approach due to a small number of positively selected sites. We considered the LRT approach to be more relevant for long and highly conserved primate sequences and therefore we used the LRT significance as the indicator of positive selection, the sliding window measure was used as a supporting score.

#### Positive selection in humans

We used the haplotype map HapMap Phase II [67] to search for the SNPs located in the gene regions (both introns and exons) of the analyzed HIV-interacting human proteins [20]. We then used the integrated haplotype score (iHS) introduced by Voight et al. [68] to identify those SNPs that might have emerged as a result of positive selection. The cutoff of |iHS| ≥2 was used to choose SNPs under positive selection. Out of 1335 SNPs in the analyzed genes 88 showed |iHS| ≥2, slightly more (6.6%) than expected from the standard normal distribution of the iHS. For each gene we calculated the number of positively selected SNPs, and used their presence as evidence of positive selection acting on a gene in the human lineage.

#### Gene Ontology

We used the R package topGO [69] to score GO terms by their overrepresentation in groups of genes. The method makes use of the hierarchical structure of the GO by first grouping genes related to each of the terms in the GO graph and then processing the nodes bottom-up. Iteratively genes annotated to significant GO terms are removed from more general parent terms to test how enriched a node is if the genes from its children nodes are not considered. We used FET to estimate the enrichment of GO terms in the subset of genes identified as being under positive selection and the KS test to test for the enrichment of the GO terms among the high-scoring genes in the gene ranking based on a positive selection score. Both tests assess term enrichment among genes under positive selection using the full set of HIV-interacting genes as a control. This helps to ensure that the significance of a term is not due to the general abundance of genes assigned to this term in the full set of HIV-interacting genes.

### Virus sequence analysis

#### Viral sequences

We searched the Los Alamos HIV sequence database [70] for complete genomes of the following viral species: HIV-1 and HIV type 2 (HIV-2), SIVcpz, rhesus macaque SIV (SIVmac) and SIVsmm. Since our primary interest was in estimating general patterns in large data sets rather than in the analysis of individual genomes, we excluded SIV species for which less than five genomes were available. In order to minimize the bias associated with the overrepresentation in the public databases of the HIVs and to obtain the highest variability of the HIV-1 genomes with approximately equal distribution among groups, we additionally filtered the HIV-1 group M genomes by retaining only therapy-naïve patient sequences, only one sequence annotated with both the same country and year and only one sequence per patient. In case of several sequences of the same year-country or patient category the longest genome sequence was selected. This filter was applied only to the HIV-1 group M sequences. All available HIV-1 group N and O complete genome sequences were kept as well as the reference HIV-1 sequence HXB2-LAI-HXB2R (accession number NC_001802). We additionally included one available *Colobus guereza *SIV (SIVcol) genome in the dataset (GenBank AF301156) as an outgroup genome for further analyses. From the complete genomes we extracted individual protein sequences according to the original genome annotation. We aligned these gene sequences using MUSCLE [71] and removed those that included more than 50% insertions or deletions as compared to the reference virus HXB2-LAI-HXB2R gene sequence. We refer to the alignment of all viruses as the full alignment. We computed an additional alignment of the HIV-1 and SIVcpz viruses only (HIV-1/SIVcpz alignment). The compiled dataset contained 76 HIV-1 genomes out of which 54 were M, 16 of type O and 6 of type N, 30 HIV-2, 19 SIVcpz, 36 SIVmac and 14 SIVsmm genomes. The filter applied to the HIV-1 group M genomes resulted in a representative diversity of the subtypes, with 35% subtype B sequences, 17% C, 8% A, one sequence of the D, E, F subtypes and 33% circulating recombinant forms of subtypes A to G. After gene extraction, alignment and filtering there were, on average, 160 sequences of each protein in the full alignment (90 in the HIV-1/SIVcpz alignment) approximately 45% of which are HIV-1 sequences (80% in the HIV-1/SIVcpz alignment).

#### Evolutionary rates

Unlike primate gene sequences, lentiviral genomes are highly variable, relatively short and contain overlapping reading frames. Given the difficulty of estimating precise selection pressures acting on such highly variable genomes, instead of using classical methods of estimating positive selection we developed a surrogate measure of *relative evolutionary rate *as follows.

Across all host species, each protein of the immunodeficiency virus has the same specific biological role in the viral life cycle and host species adaptation - a role that necessitates interaction with a set of host proteins. These roles and the resulting interactions present specific constraints and selection pressures on viral genes that contribute to the accumulation of genetic change with a rate characteristic to each viral protein. Even though the date of the introduction of the viral species into its respective host species and the duration of the infections in each host individual are unknown, the common functionality of a viral protein in different host species determines its ability to evolve at a specific rate. Therefore, we assumed that the genetic change in the viral gene relative to the genetic change in a reference gene in the same viral genome is similar among different viruses [72,73]. Using this assumption we introduced the relative evolutionary rate measure and used it to assess the rate of accumulation of genetic change in different viral protein sequences independently of the host species and the time of infection. This measure affords ranking viral genes according to the amount of genetic change accumulated among viral species.

We based the measure of relative evolutionary rate on maximum likelihood (ML) trees of nucleotide sequences estimated using the dnaml program [74], part of the PHYLIP package [75]. Due to the potential errors in dating of the SIV sequences and the difficulty of estimating evolutionary parameters with incomplete data from highly variable viral populations [72,73], more advanced phylogenetic methods [76] were not applied. We used the gamma distribution for approximating the distribution of evolutionary change at different sites with shape parameter 1 and nine rate categories [74]. These settings were selected because they resulted in the trees with the highest likelihood in several tests over a range of parameters on different viral genes.

We inferred a phylogenetic tree for each viral gene separately. Corresponding genes of the SIVcol genome were used as an outgroup in each of the trees. The distance between two gene sequences was defined as the sum of branch lengths between the nodes representing those sequences in the ML tree. Branches with low significance (p > 0.05) were excluded; pairs of sequences having such a branch between them were excluded from the calculation. Since the dataset of viral sequences was based on complete viral genomes, each of the gene trees contained corresponding sequences for each virus. We next calculated the relative evolutionary rate of each single sequence pair by dividing the distance between the two sequences of a viral gene by the distance between the reference gene sequences for the same pair of viruses. Integrase (IN) was chosen as the reference gene because its phylogenetic tree had the shortest branches on average resulting in mean relative evolutionary rates >1 for all other genes.

To account for the differing numbers of sequences of each viral species in the dataset we introduced a weighting scheme for the sequence distances to reduce the bias in the mean evolutionary rate due to the overrepresentation of the HIV-1 sequences. The weighting resulted in only minor changes in the viral gene ranking that did not influence the overall results. Thus, we chose the more parsimonious approach of not weighting.

### Gene rank correlation

In the site-based ranking we restricted the host factors to the ones under positive selection as inferred from the LRT. Both the viral and host gene rankings were normalized to the [0,1] range. The same procedure was repeated on the positively selected genes inferred in the human-chimp comparison and correlated to the viral gene ranking based on the HIV-1/SIVcpz alignment.

#### Interaction binning test

The interactions were grouped into bins defined by viral gene ranking. The ranks of host genes within bins were averaged. Bins of different sizes were slid along the viral gene ranking scale, advancing from one gene to adjacently ranked gene in each step. For each bin size we calculated the correlation between viral gene ranks and the averaged host gene ranks. Because of the small number of viral genes (18) a symmetrical approach of averaging over viral gene ranks was not performed. We tested all 18 possible bin sizes. We then used the permutation procedures described below to test for the significance of the correlation obtained for the averaged binned ranks of each bin size.

### Permutation procedures

In order to assess the statistical significance of previous analyses we developed permutation tests of the HIV-human interactions. The HIV-human interaction data can be represented by a bipartite graph with nodes representing host and viral proteins and edges connecting interacting host and viral proteins. We designed two procedures of permuting the host-virus interaction network. The *host-oriented *test consists of retaining the degree of each of the host gene nodes in the network and randomly sampling a corresponding number of interacting viral genes from the set of all viral genes. The *virus-oriented *test consists of retaining the degree of each viral gene node and randomly sampling a corresponding number of interacting host genes. Performing two different permutation tests allowed us to test if certain results were due to the differing numbers of interactions reported for different host and viral proteins. We developed additional permutation tests allowing for random node degrees in the network and found the permutation tests conserving aspects of the network topology to be more stringent in assessing the statistical significance of our observations. We therefore used the host- and virus-oriented tests to assess statistical significance of the results of our analyses.

## Authors' contributions

KB and TL conceived this project and wrote the manuscript. KB designed the study, and collected and analyzed the data. All authors read and approved the final manuscript.

## Supplementary Material

Additional file 1 **list of all analyzed genes, scores and annotations**. Click here for file

Additional file 2 **detailed results of the GO term enrichment tests**. Click here for file

Additional file 3 **information on the alignment quality**. Click here for file
