# Supplementary material for: Positive selection of HIV host factors and the evolution of lentivirus genes
Source: BMC Evol Biol. 2010 Jun 18;10:186. doi: 10.1186/1471-2148-10-186 (PMC2906474; doi:10.1186/1471-2148-10-186)
Supplement: Additional file 3 — information on the alignment quality. [file 1471-2148-10-186-S3.DOC]

**Alignment quality and its correlation with the positive selection scores**

|  | all | chimp | orangutan | macaque |
| --- | --- | --- | --- | --- |
| removed gene sequences [%] | 18.1 | 7.8 | 14.8 | 14.2 |
| mean gap ratio | 0.0183 | 0.0234 | 0.0241 | 0.0284 |
| sliding window score - gap ratio correlation | 0.0101(0.293) | 0.0021(0.473) | -0.0196(0.829) | 0.0292(0.07) |
| site-based score - gap ratio correlation | -0.0977(0.962) | -0.1123(0.984) | -0.1402(0.999) | 0.0107(0.406) |

Genes with no sequence found in the human genome were excluded from the analysis as were genes with more than one unidentified homolog among the three non-human primate species. Alignment of homologous sequences containing more than 50% gaps as compared to the human gene sequence were also excluded from the tests for positive selection. The first row of the table lists the percentage of the sequences excluded according to these criteria. The second row shows the quality of the alignment of the remaining sequences in terms of the mean ratio of the number of gaps over the length of a gene sequence. The last two rows show the correlation between the quality of alignment and the result of the positive selection tests. It appears that high scores of positive selection are not correlated with poor sequence alignment. P-values are given in parentheses.
